# A Systematic Review and Network Meta-Analysis of Randomized Controlled Trials Evaluating the Evidence Base of Melatonin, Light Exposure, Exercise, and Complementary and Alternative Medicine for Patients with Insomnia Disorder

**DOI:** 10.3390/jcm9061949

**Published:** 2020-06-22

**Authors:** Chiara Baglioni, Zarina Bostanova, Valeria Bacaro, Fee Benz, Elisabeth Hertenstein, Kai Spiegelhalder, Gerta Rücker, Lukas Frase, Dieter Riemann, Bernd Feige

**Affiliations:** 1 Department of Psychiatry and Psychotherapy, Medical Center, Faculty of Medicine, University of Freiburg, 79104 Freiburg, Germany; zarina-bostan@live.de (Z.B.); fee.benz@uniklinik-freiburg.de (F.B.); kai.spiegelhalder@uniklinik-freiburg.de (K.S.); lukas.frase@uniklinik-freiburg.de (L.F.); dieter.riemann@uniklinik-freiburg.de (D.R.); bernd.feige@uniklinik-freiburg.de (B.F.); 2 Department of Human Sciences, Guglielmo Marconi University, 00193 Rome, Italy; v.bacaro@unimarconi.it; 3 University Hospital of Psychiatry and Psychotherapy, University of Bern, 3012 Bern, Switzerland; Elisabeth.Hertenstein@upd.ch; 4 Institute of Medical Biometry and Statistics, Medical Center, Faculty of Medicine, University of Freiburg, 79104 Freiburg, Germany; ruecker@imbi.uni-freiburg.de

**Keywords:** insomnia, melatonin, meditative movement therapies, exercise, transcranial magnetic resonance, hypnotherapy, network meta-analysis

## Abstract

Insomnia is a prevalent disorder and it leads to relevant impairment in health-related quality of life. Recent clinical guidelines pointed out that Cognitive-Behavior Therapy for Insomnia (CBT-I) should be considered as first-line intervention. Nevertheless, many other interventions are commonly used by patients or have been proposed as effective for insomnia. These include melatonin, light exposure, exercise, and complementary and alternative medicine. Evaluation of comparable effectiveness of these interventions with first-line intervention for insomnia is however still lacking. We conducted a systematic review and network meta-analysis on the effects of these interventions. PubMed, PsycInfo, PsycArticles, MEDLINE, and CINAHL were systematically searched and 40 studies were included in the systematic review, while 36 were entered into the meta-analysis. Eight network meta-analyses were conducted. Findings support effectiveness of melatonin in improving sleep-onset difficulties and of meditative movement therapies for self-report sleep efficiency and severity of the insomnia disorder. Some support was observed for exercise, hypnotherapy, and transcranial magnetic resonance, but the number of studies for these interventions is still too small. None of the considered interventions received superior evidence to CBT-I, which should be more widely disseminated in primary care.

## 1. Introduction

Insomnia is one of the most frequent psychophysiological disorders [1] and it leads to clinically relevant impairment in diverse areas of health-related quality of life [2] as well as to high societal costs [3]. The Diagnostic and Statistical Manual of Mental Disorders (DSM 5) [4] defines insomnia as a difficulty in initiating/maintaining sleep or early morning awakening accompanied by decreased daytime functioning, such as fatigue/malaise, daytime sleepiness, mood disturbances/irritability, motivation/energy/initiative reduction, and attention/concentration/memory impairment, occurring at least three times per week for a period of at least 3 months. Women have an increased risk to develop insomnia compared to men [5] and the prevalence of the disorder increases significantly with age [6].

Patients with insomnia have an enhanced risk of developing mental disorders [7,8,9]. Moreover, in the last decade, research has shown that insomnia is also a risk factor for physical diseases as arterial hypertension, myocardial infarction, and chronic heart failure [10,11,12,13]. Besides insomnia itself, there is evidence suggesting that short sleep duration (sleeping less than 6 h per night on average) is an important risk factor for developing obesity, type 2 diabetes, hypertension, and cardiovascular diseases [14,15,16]. Consequently, short sleep duration also increases mortality [17]. Epidemiological data and data relating to health care as well as the cost situation of chronic insomnia reflect a huge impact on the health system and on society in general. Annual direct and indirect costs for insomnia have been estimated to be around $150 billion in the US [18], being mainly related to indirect costs such as increased health care utilization, poorer performance at work, and enhanced accident risk [19].

Even though insomnia is one of the most commonly encountered disorders in medical practice, not more than a quarter of insomnia patients seek help for their sleep problem; most of the remainder is unrecognized and medically untreated [20,21]. In 2005, the National Institutes of Health (NIH) published a State-of-the-Science Conference on manifestations and management of chronic insomnia in adults, which stated that only Cognitive-Behavior Therapy for Insomnia (CBT-I) and hypnotic medications, such as benzodiazepines (BZ) and benzodiazepine receptor agonists (BZRA), have received empirical support to be recommended. More recent guidelines in the US and Europe further pointed out that CBT-I should be the first-line treatment for the disorder [21,22,23]. However, this is available only to a tiny minority of afflicted individuals, and often used in academic environments and in the research context [21,23].

In real life, patients are often offered or self-treat their sleep problems with diverse interventions, with respect to which still few systematic information is available. For example, previous studies suggested that some forms of insomnia may be partially explained by a circadian rhythm phase delay (e.g., [24]). Thus, it has been proposed to treat sleep-initiating problems with melatonin or light exposure therapy. Melatonin and light are the strongest Zeitgeber to human circadian rhythm. Melatonin is a hormone secreted primarily by the pineal gland, located behind the third ventricle in the brain, in response to night onset as detected by specialized retinal photoreceptors. In contrast to most available sleep medications, melatonin supplementation has been shown to be safe in the short-term [25] and to have very low rebound rate [26]. Melatonin acts by altering some specific aspects of sleep architecture and consequently improving sleep quality [27]. The aim of melatonin supplementation therapy is to attain physiological control of the sleep/wake cycle. Light exposure therapy is a non-pharmacological intervention consisting in ocular exposition to bright light. Time of the day is an important factor to be manipulated in this treatment [28]. Light exposure during the evening can delay sleep onset, while light exposure in the early morning can anticipate sleep onset. Longer duration and higher intensity seem to be associated with greater effects within a certain limit, as these increases have been reported to be nonlinear [29]. Furthermore, the human circadian system is more sensitive to short-wavelength (blue) light [30], although at higher intensity no much difference is found between blue enriched light and white broad spectrum light [30]. Ocular exposition to bright light is capable of phase shifting the circadian rhythms in humans. Consequently, it has been proposed as a treatment for those disorders in which a circadian alteration is central.

Another hypothesis advanced in the literature is that sedentary lifestyle may be involved in the causation and maintenance of insomnia. Physical activity in clinical research context can include different prescriptions, including advices, counseling, provision of written material, or referral to an exercise program [31]. Regular physical exercise is considered to be a protector against different chronic somatic and mental disorders and its promotion is a priority for all health agencies [32]. Data on good sleepers showed beneficial effects of regular physical exercise on sleep quality [33].

Finally, patients with insomnia often recur to complementary and alternative medicine [34]. This refers to a set of interventions not typically included in the conventional medical care or that origin outside of usual Western practice [35]. They are defined as “alternative” when used instead of conventional medicine, and “complementary” when used together with conventional medicine. Recent classifications [36] organized different practices in five main groups: (1) alternative medical systems (including ayurvedic medicine, Chinese traditional medicine, homeopathy, Japanese traditional medicine, naturopathy, Tibetan traditional medicine); (2) natural product-based therapies (including chelation therapy, hydrotherapy, nutrition, oxygen, ozone, and herbal medicine); (3) energy therapies (including acupuncture, breathing exercises, distant healing, electric stimulation therapy, magnetic therapy, phototherapy, reiki, therapeutic touch, and ultrasonic therapy); (4) manipulative and body-based methods (including Alexander technique, chiropractic/spinal manipulation, massage, and reflexology); (5) mind–body interventions (including biofeedback, hypnosis, meditation, play therapy, relaxation techniques, sensory art therapies, tai chi, unconventional psychotherapies, and yoga). It has been estimated that ca. 1.6 million US adults use some form of CAM therapy to handle insomnia or sleep problems (NIHS, 2002). A systematic review of 20 randomized controlled trials (RCTs) published in English and using a suitable control intervention (non-active placebo or established positive control, e.g., benzodiazepine) evaluated complementary and alternative interventions (CAI) for insomnia, including herbal and nutritional medicine, acupuncture, acupressure, yoga, tai chi, massage, aromatherapy and homoeopathy [37]. The authors pointed out the relative weakness of the examined literature, involving small sample sizes and/or short treatments durations. Some positive findings are reported for acupuncture, but literature is mixed and inconsistent. This qualitative note is consistent with a Cochrane meta-analysis on acupuncture in insomnia that found clinical trials had effect sizes generally small and inconclusive [38,39].

With respect to these mentioned interventions, recent European guidelines for insomnia [23] disorder state the following.

1. Melatonin is generally not recommended because evidence shows low efficacy.
2. Light therapy and physical activity may be useful as adjunct therapy, but recommendation is weak due to low-quality evidence.
3. Complementary and alternative interventions are not recommended because of poor evidence. In the area of complementary and alternative interventions, several treatments for insomnia have been suggested, including acupuncture, acupressure, aromatherapy, reflexology, homeopathy, meditative movement therapies, moxibustion, music therapy, and yoga [40].

Updating systematic evaluation of comparable efficacy of these treatments is needed for two reasons. First, as patients often recur to these therapies, evidence-based data and comparisons with current recommended interventions is a priority. Second, although the efficacy of CBT-I is well proved (for a review see [23]), still many patients retain sleep disturbance after CBT-I (see, e.g., in [41]). Thus, it is necessary to identify treatments which may be alternative to CBT-I for those patients who do not respond to the main clinical procedures.

The aim of the present work was to provide a systematic review on the comparable efficacy of melatonin, light exposure therapy, exercise, and complementary and alternative medicine for insomnia disorder with respect to CBT-I, hypnotic medication or placebo/waiting list control conditions. We considered all age ranges including adult and pediatric populations. Acupuncture/acupressure was not included in our list, because of the extensive work previously published through the Cochrane Library [38,39].

In the context of evidence-based medicine, network meta-analysis aims to compare a number of available treatments for a given diagnosis by combining direct and indirect evidence on treatment effects based on a common comparator [42,43]. Network meta-analysis is a valid statistical method which allows for simultaneous analysis of both direct and indirect comparisons among multiple treatments across multiple studies. This method has advantages over pairwise meta-analysis which allows comparison of only two interventions, including (a) borrowing strength from indirect evidence to compare all treatments, (b) estimating comparative effects that have not been investigated head-to-head in randomized clinical trials, and (c) comparing between more than two interventions for one condition which informs clinical practice. To explain the concept underpinning network meta-analysis, and specifically the meaning of direct and indirect evidence, we suppose we compare two active treatments A and B and a control condition C. Given direct evidence from studies regarding the difference of treatment effects for A and C and evidence regarding the difference of treatment effects for B and C from other studies, these studies also provide indirect evidence for treatments A and B. If there is direct evidence for A vs. B, direct and indirect evidences are combined to get the most precise estimate of treatment difference. Therefore, the aim of network meta-analysis is to estimate the treatment differences and associated standard errors combining direct and indirect evidence [44].

## 2. Methods

The present study was conducted according to the Preferred Reporting Items for Systematic reviews and Meta-Analyses (PRISMA) guidelines for systematic review incorporating a network meta-analysis [45] (see Document S1: PRISMA check-list). The study protocol was reviewed and approved for funding from the German Ministry for Education and Research (Bundesministerium für Bildung und Forschung, BMBF, 01KG1111).

### 2.1. Search Procedure

We used several strategies to identify our final study sample.

First, we conducted computer-based searches using PubMed, PsycInfo, PsycArticles, MEDLINE, and CINAHL using the following keywords, capturing the title and the abstract: ‘insomnia’ AND ‘ayurveda’ OR ‘chelation’ OR ‘diet based therapy’ OR ‘energy healing therapy’ OR ‘exercise’ OR ‘folk medicine’ OR ‘homeopathic’ OR ‘hypnosis’ OR ‘light exposure’ OR ‘massage’ OR ‘meditation’ OR ‘melatonin’ OR ‘music therapy’ OR ‘natural herbs’ OR ‘naturopathy’ OR ‘qi gong’ OR ‘reiki’ OR ‘tai chi’ OR ‘transcranial magnetic stimulation’ OR ‘valerian’ OR ‘vitamin’ OR ‘yoga’. The search was conducted from January 1994, the date in which the DSM-IV [46] was published and September 2019. All abstracts found through keywords search were saved in a project in CITAVI software. CITAVI is a software for references management and organization (https://www.citavi.com). All abstracts were screened by four independent raters coordinated by the second author (Z.B.) collaborating together with the first author (C.B.) whenever the inclusion or exclusion of one study was doubtful. Similar procedures were followed for full-text screening.

Second, we expanded our search through identifying further studies from the references of the screened full-texts.

Third, we contacted authors in the field through the mailing list of the European Insomnia Network (a group of experts in insomnia promoted by the European Sleep Research Society (ESRS)), to obtain further published studies and, if needed, to obtain additional information.

Fourth, unpublished data were searched through contact with experts in the field (as described in the previous paragraph) and through screening of all abstracts of poster and oral presentations to the Conference of the European Sleep Research Society of 2014, 2016, and 2018 and to the Conferences of the American Associate Professionals Sleep Societies of 2014, 2015, 2016, 2017, and 2018.

### 2.2. Eligibility Criteria

We included all clinical studies evaluating melatonin, light exposure therapy, exercise, and complementary and alternative medicine interventions for insomnia based on the following eligibility criteria and the Population, Intervention, Comparison, Outcomes and Study design (PICOS) approach [45]. PICOS is a structured approach for framing questions which assess five components and may help facilitate the process: the patient population or the disease being addressed (P); the interventions of exposure (I); the comparator group (C); the outcome or endpoint (O); and study design (S):

(1) Population: Individuals with insomnia disorder of all ages (including adult and pediatric populations) and of both gender with or without any mental, somatic or sleep comorbidity.
(2) Intervention: Experimental interventions including one of the following administered alone (i.e., not in combination with recommended therapies for insomnia): ayurveda, chelation, diet-based therapy, energy healing therapy, exercise, folk medicine, homeopathy, hypnosis, light exposure, massage, meditation, melatonin, music therapy, natural herbs, naturopathy, qi gong, reiki, tai chi, transcranial magnetic stimulation, valerian, vitamin, and yoga.
(3) Comparison: waiting list, no treatment, pharmacological and psychological (e.g., psychoeducation) placebo, standard therapy for insomnia: sleep pharmacotherapy (hypnotics: benzodiazepine (BZ) and benzodiazepine receptor agonists (BZRA) and recommended psychological treatment, i.e., CBT-I (CBT-I, sleep restriction, stimulus control).
(4) Outcomes: objective and subjective standardized measures of sleep and/or insomnia.
(5) Study design: Randomized controlled trial.
(6) Diagnosis of insomnia based on DSM-IV/DSM-IV-TR/DSM-5 [4,46,47], or on the International Classification of Sleep Disorders second and third versions [48,49] or consistent with those definitions
(7) Written in English, German, Italian, Spanish, French, Bulgarian, or Russian.

### 2.3. Data Extraction

Descriptive information related to sample demographic and clinical characteristics, clinical aspects of insomnia disorder, intervention characteristics and methodological aspects of the study design was collected. Descriptive information included (a) age and sex of participants in the experimental and control interventions; (b) diagnostic tool/s according to which participants met the definition of insomnia disorder; (c) past medical conditions; (d) information about mental and somatic comorbidities; (e) number of treatment sessions and duration; (f) treatment setting and conducting personnel; (g) type of control intervention; (h) insomnia duration.

After data extraction (see results), treatments were grouped in subgroups of interventions: (1) Meditative movement therapies: the cluster included meditation, mindfulness, tai chi, yoga; (2) Dietary supplements; (3) Melatonin; (4) Light exposure; (5) Natural herbal pharmacotherapies: the cluster included valerian, natural herbs, naturopathy; (6) Exercise; (7) Transcranial magnetic resonance (rTMS); (8) Homeopathy; (9) Hypnotherapy; (10) Placebo/Waiting list: this cluster included control conditions such as pharmaceutical placebo, self-monitoring, no treatment, usual lifestyle, and waiting list; (11) Cognitive Behavior Treatment for Insomnia (CBT-I); (12) Pharmacotherapy. *Outcome* measures included both self-report and physiological indices of sleep parameters, sleep quality and insomnia, measured through sleep diaries, questionnaires, polysomnographic recordings and/or actigraphy.

### 2.4. Assessment of Risk of Bias

Methodological variables were assessed through the Cochrane Collaboration’s tool for assessing risk of bias in randomized controlled trials [50]. In this tool five main domains are assessed with a judgement of high, low or unclear risk.

(1) Selection bias. This domain refers to systematic differences between baseline characteristics and covers two parts: (1) Did the investigators use a random sequence generation process? (2) Could intervention allocations have been foreseen in advance of enrolment?
(2) Performance bias. This domain judges whether participants and personnel were blinded. Because blinding therapists and patients is not desirable in some form of interventional studies (such as psychotherapy or mindfulness or yoga), performance biases for these types of studies was systematically scored as “low risk”.
(3) Detection bias. This domain refers to whether outcome assessors are aware of intervention assignments.
(4) Attrition bias. This domain refers to systematic differences between groups in withdrawals from a study. Amount, nature, and handling of incomplete outcome data are evaluated.
(5) Reporting bias. This domain refers to selective outcome reporting. For each included clinical study a search was conducted to find registered protocols in order to check the consistency between the planned and the reported analyses.

Each study was rated by two independent raters (Z.B. and V.B.) as at low risk (1), moderate risk (2), and high risk (3) with respect to five areas of methodological risk: randomization and allocation methods (selection bias), blinding of participants and personnel (performance bias) and of outcome assessors (detection bias), incomplete outcome data (attrition bias), and selective reporting (reporting bias). For selection bias, two different scores were applied for randomization and allocation methods. Divergences were discussed with the first (C.B.) and the fourth (F.B.) authors.

### 2.5. Statistical Analyses

Analyses were planned for an application for funding by the German Ministry for Education and Research (Bundesministerium für Bildung und Forschung, BMBF, 01KG1111). The plan was structured together with a biostatistician with excellent expertise in network meta-analyses (G.R.).

Data were extracted both for sleep and insomnia related variables as well as for daytime symptoms. Data are available in the online Supplementary Materials online (Document S2: Data extraction). Nevertheless, neither statistical analysis nor qualitative summary was conducted for data related to daytime symptoms, as these were too few and too different between studies.

Eight network meta-analyses were conducted considering experimental and control interventions categories as explained above (Data extraction) for the following categories of outcomes.

(1) **Self-reported sleep efficiency**: defined as a sleep efficiency index from sleep diaries or, if this was not reported, as sleep quality perception from sleep diaries;
(2) **Sleep efficiency measured through physiological indices**: defined as sleep efficiency index measured by polysomnography, or, if this was not reported, by actigraphy;
(3) **Self-reported daytime sleepiness**: defined as total score of the Epworth Sleepiness Scale [51], or, if this was not available, of the Children’s Sleep Habits Scale (daytime symptoms which includes a scale of daytime sleepiness [52]);
(4) **Self-reported severity of sleep problems**: defined as total score from Insomnia Severity Index [53], or, if this was not available, from the Pittsburgh Sleep Quality Index [54].
(5) **Self-reported sleep onset latency**: defined as sleep onset latency measured through sleep diaries;
(6) **Sleep onset latency measured through physiological indices**: defined as sleep onset latency measured by polysomnography or, if this was not reported, by actigraphy.
(7) **Self-reported wake time during the night**: defined as wake after sleep onset latency measured through sleep diaries;
(8) **Wake time during the night measured through physiological indices**: defined as wake after sleep onset latency measured by polysomnography or, if this was not reported, by actigraphy.

Meta-analytic calculations were performed with the statistical software package R [55] (http://www.Rproject.org/). A frequentist network meta-analysis was performed using the R-package “netmeta” [56,57]. Through network meta-analysis it is possible to graphically represent the comparisons included in the network. The function “netgraph” was used to visualize a net-graph evidencing the geometry of the network. The network graph consists of nodes that represent the treatments and edges that represent direct evidence, i.e., treatments that are directly compared in at least one study. The width of those lines is based on sample size for those comparisons. Finally, shadows indicate multi-arm studies.

Second, we calculated effect sizes as standardized mean differences (Cohens’d) between post-treatment assessment and baseline. We referred to data of participants who completed the post-treatment assessment. All classes of interventions were compared against placebo/waiting list interventions because this category of control treatment was used most frequently. A random-effects model was used because of expected considerable heterogeneity between studies (e.g., comorbidities, different treatment variables, and different populations).

To test the heterogeneity of the network, Cochran’s Q and Higgin’s I^2^ were calculated. Cochran’s Q is computed as a weighted sum of squared differences between single study effects and the pooled effect across studies. Significant values indicate a high level of heterogeneity between studies that needs to be further investigated. Higgin’s I^2^ calculates the variability in effect estimates that is due to between-study heterogeneity rather than due to chance. Low percentages of I^2^ are indicative of low heterogeneity while percentages over 75% represent considerable levels of heterogeneity [58].

In network meta-analyses, net heat plots can be plotted to investigate potential sources of heterogeneity [59]. These are graphical tools that represent changes in heterogeneity due to relaxing the consistency assumption for single designs in a matrix visualization. In the matrix, areas of the grey squares indicate the contribution of pooled direct evidence of each single design in the column to each network estimate in the row. This tool also graphically represents changes in Cochran’s Q statistic due to relaxing the consistency assumption for single designs in matrix visualization. “Hot spots” of inconsistency are indicated by red colors while blue colors indicate consistency.

## 3. Results

### 3.1. Study Selection

**Figure 1:**
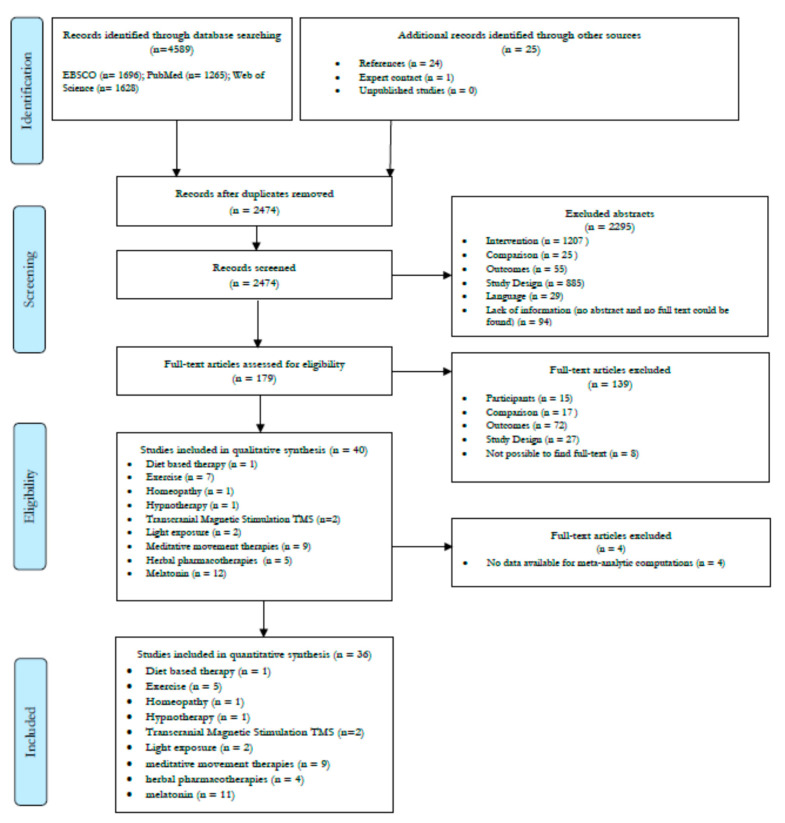
Search flow.

Figure 1 illustrates the search flow of the studies included in the present systematic review and network meta-analysis. Literature search between January 1994 – September 2019 yielded 4589 abstracts (EBSCO n= 1686; PubMed n= 1265; Web of Science n= 1628). After removing duplicates and adding articles found through search in other sources a total of 2474 studies were found. Of these, 40 studies were included in the systematic review while 36 were entered in the network meta-analysis (four studies were excluded because data were not available). Details are shown in Figure 1.

### 3.2. Study Characteristics

No study could be selected for ayurveda, chelation, energy healing therapy, folk medicine, reiki, vitamin, massage, music therapy, and qi gong. Of the 40 studies selected, 12 focused on melatonin as possible intervention for insomnia disorder [60,61,62,63,64,65,66,67,68,69,70,71], 9 on meditative movement therapies [72,73,74,75,76,77,78,79,80] (particularly, n = 1 on yoga [72], 6 on mindfulness [73,74,75,78,79,80] and 2 on tai chi [76.77]), 7 on exercise [81,82,83,84,85,86,87], 5 on natural herbal pharmacotherapy [88,89,90,91,92], 2 on light exposure [93,94], 2 on transcranial magnetic stimulation (rTMS) [95,96], 1 on homeopathy [97], 1 on hypnotherapy [98], and 1 on dietary supplements [99]. Two studies which evaluated exercise [82,83], one study evaluating melatonin [61], and one study evaluating natural herbal pharmacotherapy [88] were not included in the meta-analysis as complete data were not available. Control conditions of the included studies were waiting list (n = 11), placebo (n = 23), pharmacotherapy (n = 3), and CBT (n = 3).

Included studies focused on different age categories, specifically: 5 studies focused on children and adolescents (<18 years) [60,61,62,67,68], 23 studies focused on working-age adults (18–65 years) [70,71,72,73,75,78,79,81,82,83,84,86,87,89,90,91,92,94,95,96,97,98,99], and 2 studies focused on elderly (>65 years) [65,80]. Ten studies used mixed age samples with adult and elderly participants [63,64,66,69,74,76,77,85,87,93]. One study was composed of only male participants [86] and two studies of only female participants [72,73], while all other studies recruited mixed samples of both female and male participants. Seven studies excluded participants with past medical disorders [63,69,71,82,86,89,95]; 15 of the selected studies excluded participants with other mental disorders than insomnia [63,65,69,71,74,80,82,83,84,85,86,87,89,93,99]; eight studies excluded participants with comorbid medical disorders [64,65,71,89,91,95,98,99]; and 27 studies excluded participants with comorbid other sleep disorders than insomnia [60,62,63,64,65,66,67,69,70,71,73,74,75,78,79,83,85,86,87,89,90,91,92,93,94,96,99].

For psychological/behavioral interventions, number of sessions ranged from a minimum of 1 [84] to a maximum of 117 [86], with a range duration of the all experiments from a minimum of 1 day [84], to a maximum of 26 weeks [86]. For other interventions duration ranged from a minimum of 1 week with a dose of once a day [94], to a maximum of 6 months with a dose of once a day [64].

Twenty studies referred to the Diagnostic and Statistical Manual of Mental Disorder (n = 18, DSM IV; DSM IV TR; DSM-5) [62,63,65,66,68,70,71,72,73,80,81,86,87,90,91,95,96,98] or the International statistical classification of diseases and related health problems (n = 2, ICD-10) [88,97] for the diagnosis of insomnia, two studies used International Classification of Sleep Disorders (ICSD) [92,93], three used the Research Diagnostic Criteria for Insomnia Disorder [78,82,83] and eight studies used more than one of these categorizations together [69,76,77,78,79,82,84,89]. Of the remaining studies, seven used criteria consistent with DSM-5 categorization [60,62,64,67,85,89,94].

Average insomnia duration ranged from 1 month to 11.3 years, fifteen studies did not report this information. Descriptive information on sample characteristics and outcome measure is reported in Table 1. Further information on study characteristics is reported in the online supplemental material Document S3: Study characteristics.

### 3.3. Risk of Bias

**Figure 2:**
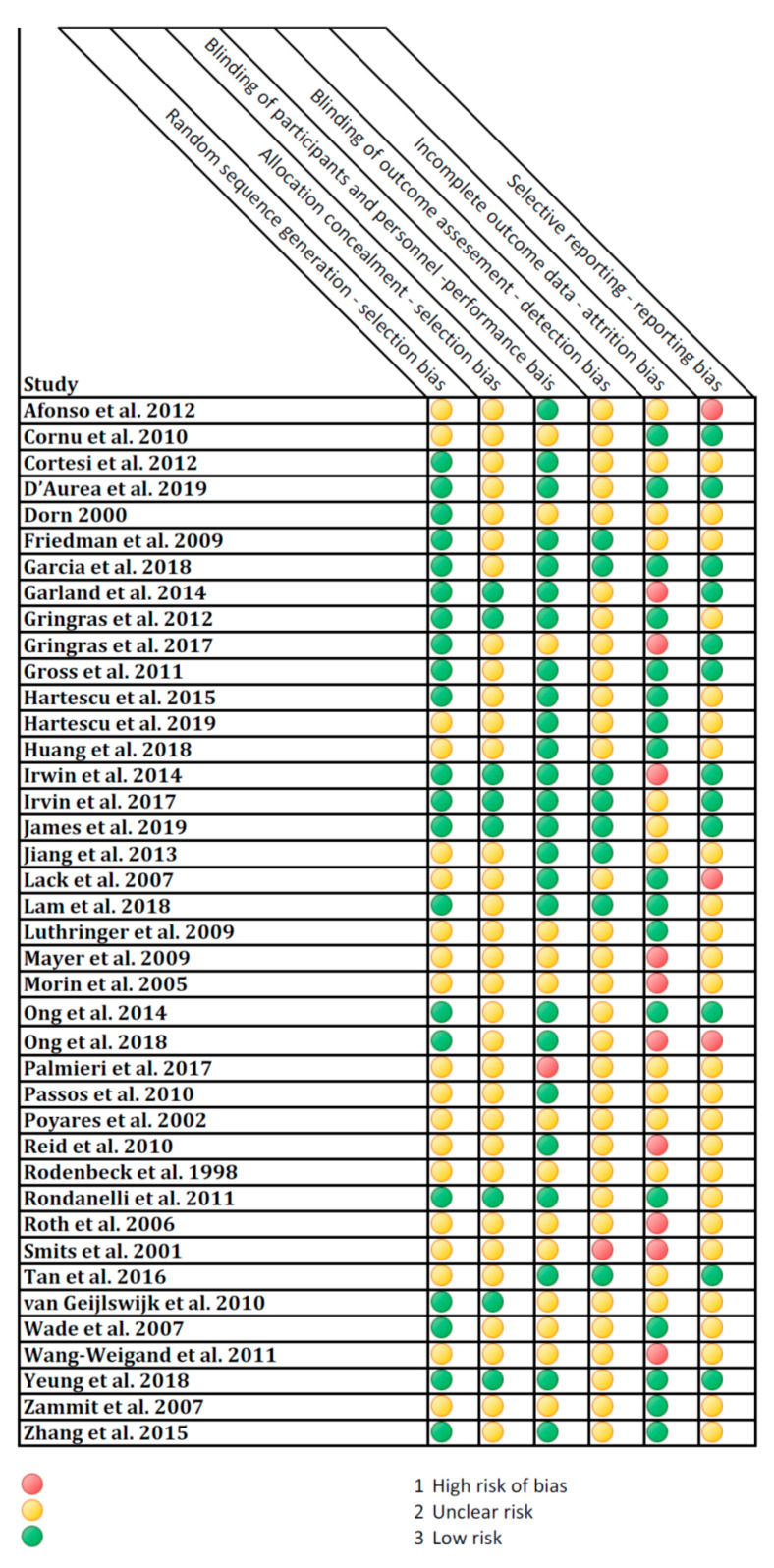
Risk of bias.

Figure 2 illustrates the studies’ evaluation for risk of bias. In supplemental material online Document S4 the risk of bias evaluation is reported in detail. Only two studies received two “High risk” judgments [67,79] in detection bias and attrition bias and selective reporting, and not a single study received all “Low risk” judgment. Furthermore, two studies received all “Unclear” risk judgment [91,92].

### 3.4. Network Meta-Analysis

Eight network meta-analyses were conducted considering four categories of outcomes (explained in the description of data-analyses): (1) Self-reported sleep efficiency; (2) Sleep efficiency measured through physiological indices; (3) Self-reported sleepiness; (4) Self-reported severity of sleep problems; (5) Self-reported sleep onset latency; (6) Sleep onset latency measured through physiological indices; (7) Self-reported wake time during the night; (8) Wake time during the night measured through physiological indices. Extracted data are given in Document S2: Data extraction.

### 3.5. Self-Reported Sleep Efficiency

Sixteen studies measured subjective sleep quality.

**Figure 3:**
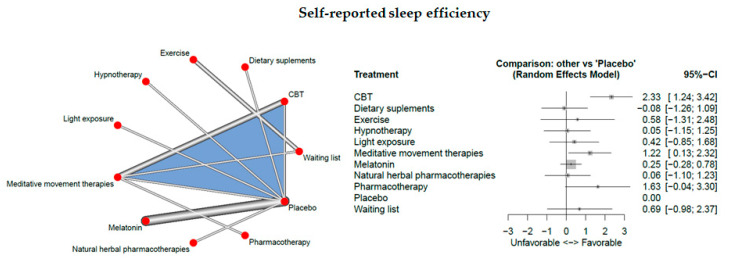
Self-reported sleep efficiency.

The network consisted of 11 interventions including experimental and control conditions, based on 18 pairwise comparisons and 10 different designs. Figure 3 presents the netgraph and the forest plot presenting the effects of all available classes compared with placebo/waiting list.

A large significant effect could be detected for CBT-I (d = 2.33, 95% CI:[1.24; 3.42]) and a significant result was observed for meditative movement therapies (d = 1.22, 95% CI:[0.13; 2.32]). No significant result was observed for the other interventions.

Cochran’s Q and Higgins I^2^ tests revealed very high heterogeneity between studies (tau^^2^ = 0.3396; I^^2^ = 95.7%). The net heat plot (see Supplementary Materials online Document S5) suggests that the designs that are together responsible for the majority of between-design heterogeneity are the following ones; (1) CBT-I vs. meditative movement therapies, (2) CBT-I vs. meditative movement therapies which is involved in a three-arm study: CBT-I, meditative movement therapies, and Placebo. Furthermore, the net heat plot suggests that the evidence for the treatment comparison CBT-I vs. meditative movement therapies from the designs CBT-I vs. meditative movement therapies (design from the three-arm study: CBT-I, meditative movement therapies, and Placebo) and CBT-I vs. Placebo (design from the three-arm study: CBT-I, meditative movement therapies, and Placebo) is inconsistent with the other evidence.

### 3.6. Sleep Efficiency Measured Through Physiological Indices

Eighteen studies measured sleep quality through physiological indices

**Figure 4:**
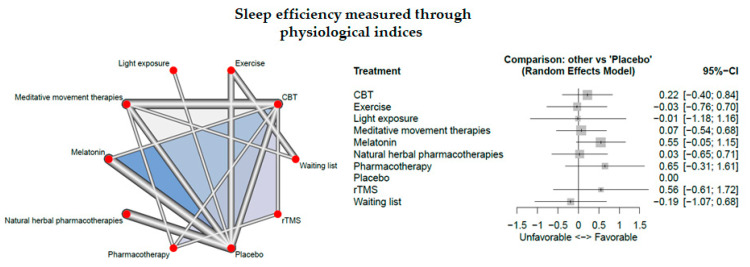
Sleep efficiency measured through physiological indices.

The network consisted of 10 interventions including experimental and control conditions, based on 24 pairwise comparisons and 12 different designs. Figure 4 presents the netgraph and the forest plot presenting the effects of all available classes compared with placebo.

No significant result could be detected.

Cochran’s Q and Higgins I^2^ tests revealed very high heterogeneity between studies (tau^^2^ = 0.2759; I^2 = 87.9%). The net heat plot (see Document S5) suggests that the designs that are together responsible for the majority of between-design heterogeneity are the following ones; (1) CBT-I vs. melatonin which is involved in a three-arm study, (2) CBT-I vs. placebo which is involved in a three-arm study and (3) Melatonin vs. placebo. Moreover, the net heat plot indicates “hot spots” of inconsistency (see the designs in red color that are not on the top-left to bottom-right diagonal, e.g., the evidence for the treatment comparison “melatonin vs. placebo” from the designs “Melatonin vs. placebo” and “CBT-I vs. melatonin” from the three-arm study “CBT-I, melatonin, placebo” is inconsistent with the other evidence).

### 3.7. Subjective Severity of the Sleep Problem

Eighteen studies measured the subjective severity of the sleep problem.

**Figure 5:**
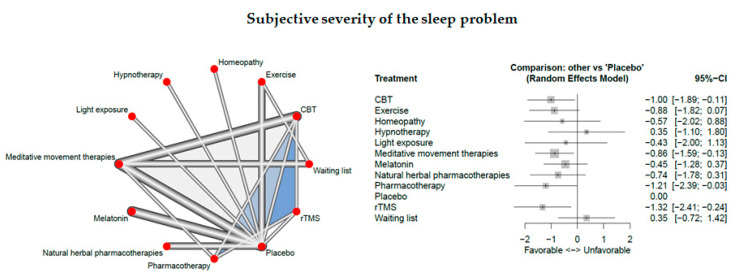
Subjective severity of the sleep problem.

The network consisted of 12 interventions including experimental and control conditions, based on 25 pairwise comparisons and 14 different designs. Figure 5 presents the netgraph and the forest plot presenting the effects of all available classes compared with placebo.

Significant results could be detected for CBT-I (d =- 1.00, 95% CI: [ −1.89; −0.11]), meditative movement therapies (d =- 0.86, 95% CI: [ −1.59; −0.13]), rTMS (d =- 1.32, 95% CI: [ −2.41; −0.24]), and pharmacotherapy (d =- 1.21, 95% CI: [ −2.39; −0.03]).

Cochran’s Q and Higgins I^2^ tests revealed very high heterogeneity between studies (tau^^2^ = 0.5109; I^2^ = 94.4%). The net heat plot (see Document S5) suggests that the designs that are together responsible for the majority of between-design heterogeneity are the following ones; (1) meditative movement therapies vs. placebo, (2) exercise vs. waitlist, and (3) Placebo vs. rTMS. Again, the net heat plot indicates “hot spots” of inconsistency (see the designs in red color that are not on the top-left to bottom-right diagonal).

### 3.8. Daytime Sleepiness

Six studies measured daytime sleepiness.

**Figure 6:**
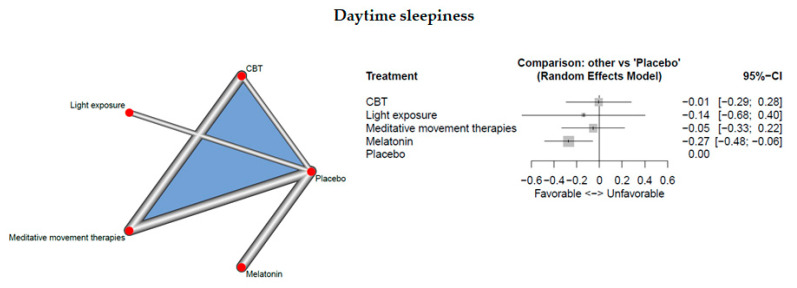
Daytime sleepiness.

The network consisted of 5 interventions including experimental and control condition, based on 8 pairwise comparisons and 5 different designs. Figure 6 presents the netgraph and the forest plot presenting the effects of all available classes compared with placebo.

Significant results could be detected for melatonin only (d = −0.27, 95% CI: [ −0.48; −0.06]). Cochran’s Q and Higgins I^2^ tests revealed low heterogeneity between studies (tau^^2^ =0; I^2 = 0%). The net heat plot (see Document S5) shows no relevant between-design heterogeneity and does not indicate “hot spots” of inconsistency.

### 3.9. Self-Reported Sleep Onset Latency

Fourteen studies measured self-reported sleep onset latency.

**Figure 7:**
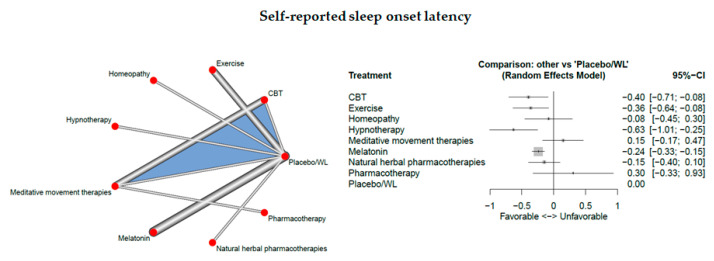
Self-reported sleep onset latency.

The network consisted of 9 interventions including experimental and control condition, based on 16 pairwise comparisons and 8 different designs. Figure 7 presents the netgraph and the forest plot presenting the effects of all available classes compared with placebo.

Significant results could be detected for CBT-I (d = −0.40, 95% CI: [ −0.71; −0.08]), exercise (d = −0.36, 95% CI: [ −0.64; −0.08]), hypnotherapy (d = −0.63, 95% CI: [ −1.01; −0.25]), and melatonin (d = −0.24, 95% CI: [ −0.33; −0.15]). Cochran’s Q and Higgins I^2^ tests revealed low heterogeneity between studies (tau^2 =0; I^2^ = 0%). The net heat plot (see Document S5) shows no relevant between-design heterogeneity and doesn’t indicate “hot spots” of inconsistency.

### 3.10. Sleep Onset Latency Measured Through Physiological Indices

Nineteen studies measured sleep onset latency through physiological indices.

**Figure 8:**
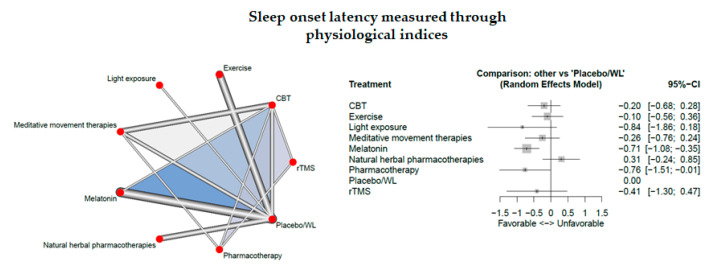
Sleep onset latency measured through physiological indices.

The network consisted of 9 interventions including experimental and control conditions, based on 25 pairwise comparisons and 10 different designs. Figure 8 presents the netgraph and the forest plot presenting the effects of all available classes compared with placebo.

Significant results could be detected for melatonin (d = −0.71, 95% CI: [ −1.08; −0.35]).

Cochran’s Q and Higgins I^2^ tests revealed high heterogeneity between studies (tau^2 =0.1446; I^2^ = 78.8%). The net heat plot (see Document S5) suggests that the designs that are together responsible for the majority of between-design heterogeneity are the following ones; (1) Meditative movement therapies vs. placebo/waitlist, (2) CBT:Placebo/WL (from the three-arm study: CBT:Meditative:Placebo/WL) and (3) CBT:Placebo/WL (from the three-arm study CBT:Melatonin:Placebo/WL). There are no “hot spots” of inconsistency.

### 3.11. Self-Reported Wake During the Night

Eight studies measured self-reported wake during the night.

**Figure 9:**
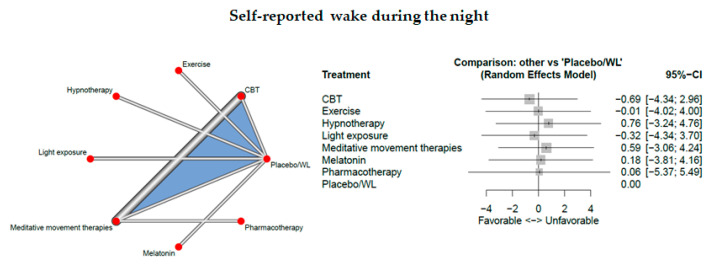
Self-reported wake during the night.

The network consisted of 8 interventions including experimental and control condition, based on 10 pairwise comparisons and 7 different designs. Figure 9 presents the netgraph and the forest plot presenting the effects of all available classes compared with placebo.

No significant results could be observed. Cochran’s Q and Higgins I2 tests revealed very high heterogeneity between studies (tau^2 =4.1301; I^2 = 99.4%). The net heat plot (see Document S5) suggests that the designs that are together responsible for the majority of between-design heterogeneity are the following ones; (1) CBT-I vs. meditative movement therapies and (2) CBT vs. Meditative (from the three-arm study: CBT:Meditative:Placebo/WL).

### 3.12. Wake during the Night Measured Through Physiological Indices

Fourteen studies measured wake during the night measured through physiological indices.

**Figure 10:**
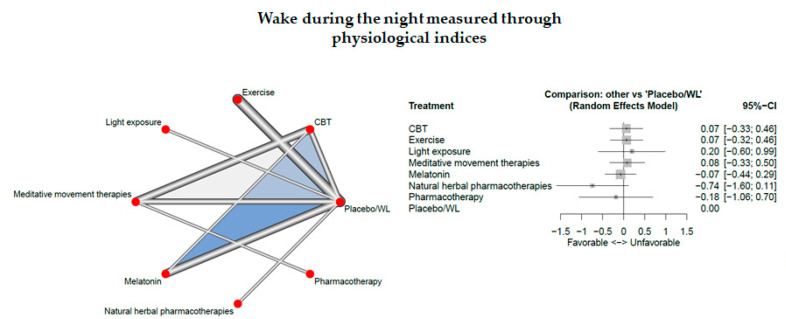
Wake during the night measured through physiological indices.

The network consisted of 8 interventions including experimental and control condition, based on 18 pairwise comparisons and 9 different designs. Figure 10 presents the netgraph and the forest plot presenting the effects of all available classes compared with placebo.

No significant results could be observed. Cochran’s Q and Higgins I^2^ tests revealed high heterogeneity between studies (tau^2 = 0.0848; I^2^ = 71.0%). The net heat plot (see Document S5) suggests that the designs that are together responsible for the majority of between-design heterogeneity are the following ones; (1) CBT vs. Melatonin (from the three-arm study CBT:Melatonin:Placebo/WL) and (2) CBT vs. Placebo/WL (from the three-arm study CBT:Melatonin:Placebo/WL).

## 4. Discussion

In the present study, network meta-analysis was used to compare the efficacy of different interventions for insomnia disorder, which have been suggested as effective, but for which clinical guidelines do not give recommendations. Specifically, the focus of this study was to compare the effectiveness of melatonin, light exposure, exercise and complementary and alternative medicine for insomnia disorder. Within this last category, many different interventions were searched, but for most of them no study was identified and selected for the systematic review and the network meta-analysis. Thus, it was possible to compare the following groups of interventions; melatonin, meditative movements therapies, exercise, natural herbal pharmacotherapies, light exposure, transcranial magnetic resonance, homeopathy, hypnotherapy, and dietary supplement.

Melatonin was evaluated in the largest group of studies in our network meta-analysis (12 of 36). Results support melatonin as an effective intervention to ameliorate sleep onset latency, both measured with physiological (i.e., polysomnography or actigraphy) and self-report (i.e., sleep diaries or questionnaires) indices, with a large effect for the physiological indices, but a rather small effect for the self-report ones. Moreover, melatonin was found to be effective in improving daytime sleepiness, although the observed effect was rather small. Nevertheless, consumption of melatonin was not associated with improved self-report or physiological measured sleep quality, perceived severity of sleep problem, and self-report or physiological measured wake during the night. Interestingly, the only five studies based on pediatric populations all tested the efficacy of melatonin. Although no group analysis on age groups was possible to conduct due to scarcity of data, this work suggests that melatonin could be effective for sleep-onset problems in both pediatric and adult populations with specific problems in initiating sleep. For children, use of melatonin has received better clinical favor [100]. As melatonin was not effective considering sleep-efficiency index and severity of the disorder, it is likely that the use of melatonin should be combined with CBT-I to obtain significant clinical benefits. The study conducted by Cortesi and colleagues [60] provides some preliminary evidence that combining behavioral treatment for insomnia with melatonin in children (age range: 4–10 years) may be more effective than melatonin or behavioral intervention only. Nevertheless, more research on this is needed.

Meditative movement therapies were evaluated in nine studies. Results showed large effects supporting the offer of these therapies for ameliorating perceived sleep efficiency and severity of the sleep problem. Instead, no support was evidenced for the other measured variables. Comparability data did not evidence superior effects for meditative movement therapies with respect to CBT for insomnia. These results suggest that meditative movement therapies may be important as additional interventions or strategies in the treatment of insomnia, but that they may be not adequate to be offered alone. Meditative movement therapies mainly target emotion regulation processes. The role of emotional processes in the consolidation of the disorder of insomnia has been stressed [101]. The introduction of mindfulness modules in the standard protocol for CBT-I has been already used in many recent large clinical trials [102]. It would be interesting to test whether these interventions targeting emotion regulation and self-regulation are specifically effective for daytime symptoms. As sleep is strictly associated with socioemotional development [103], it seems to be important to evaluate combined therapies including CBT-I and meditative movement therapies in children and adolescents.

Exercise and hypnotherapy resulted efficacious in ameliorating self-reported sleep onset latency with small to medium effects. Nevertheless, only two studies were available for exercise, including in total 61 adults with insomnia disorder randomized to experimental intervention (N = 30) or control condition (N = 31). Hypnotherapy was tested in one single study of our sample, including 60 adults with insomnia randomized to experimental (N = 30) or control (N = 30) conditions. Similarly, transcranial magnetic resonance was observed to have a large positive effect for perceived severity of the sleep problem, but was also tested in only two studies including in total 116 adults with insomnia disorder randomized to experimental intervention (N = 58) or control condition (N = 58). Thus, no strong conclusions or hypotheses may be driven by these results.

No supportive evidence for natural herbal pharmacotherapies, light exposure, homeopathy, and dietary supplement was observed.

High to very high heterogeneity was found for all outcomes with the exception of self-reported sleep onset latency and daytime sleepiness. Therefore, results must be interpreted with caution.

Some limitations of the present work should be considered. Some interventions have been considered in a larger number of studies compared to others. Especially, most studies focused on melatonin and meditative movement therapies, whereas other therapies were considered in a relative small number of trials. That resulted in networks with unbalanced designs. Quality of the study is questionable, as no study was included for which a total judgment of “low risk” could be applied. It is strongly encouraged that clinical trials evaluating possible alternative interventions for insomnia disorder to those recommended carefully consider all possible biases which may limit interpretation of the results. Moreover, subgroup analyses were not conducted because data were too few for many of the considered interventions and study characteristics were too different between trials. Furthermore, diversity in studies clinical procedures and populations was extremely high. Another limitation is that publication bias could not be analyzed. It is still debated in literature how to conduct publication bias analyses in the specific context of network meta-analyses. Funnel plots which are commonly used in traditional meta-analyses to assess publication bias are not recommended with regard to network meta-analyses where the direction of effects of small studies cannot be assumed. That is, the results presented in this work may be susceptible to the publication bias, thus their interpretation should be done carefully. Finally, no follow-up data could be summarized in the network meta-analysis, due to excessive diversity of study designs.

## 5. Clinical Implications

The results of our network meta-analysis do not evidence any of the considered interventions to be effective for both nighttime sleep symptoms and perception of severity of insomnia. Thus, our findings do not support any of the selected therapies to be recommended for insomnia disorder. Instead, our work supports the notion that much more should be done to offer first-line treatment of insomnia, i.e., cognitive-behavioral therapy (CBT), in primary care. Interesting findings associated with the use of melatonin, meditative movement therapies, and exercise should be considered. Future research should be conducted to assess the efficacy of protocols combining melatonin and CBT for insomnia for those patients who mostly complain of sleep onset difficulties. In addition, more trials are encouraged to evaluate the efficacy of protocols combining standard CBT for insomnia with modules including meditative movement therapies or exercise. This should be done by considering efficacy for both nighttime and daytime symptoms.

## 6. Conclusions

The results of this study do not support other interventions to replace or to be comparable to the first-line treatment of insomnia (i.e., CBT for insomnia disorder). As a consequence, it is considered necessary that much more effort is done to better implement and disseminate CBT for insomnia in primary care. A recent paper of the European Academy for Cognitive-Behavioral Therapy for Insomnia (CBT for insomnia) has provided guidelines for improving clinician’s training and therapy dissemination [21]. Some important hypotheses should be further tested, considering both nighttime and daytime symptoms. These would be specifically directed to consider the role of melatonin as an integrative treatment for patients with severe sleep onset difficulties, and to consider the role of meditative movement therapies and exercise as integrative therapies for daytime symptoms and perception of sleep quality and severity of the sleep problem. A lifespan approach considering pediatric, adult, and elderly populations should be encouraged.

## Figures and Tables

**Table 1:** Study characteristics.

Study	Intervention of Interest	Intervention of Reference	Age Range	Sex (% of Females)	Comorbidities	Insomnia Definition	Sleep/Insomnia Outcomes
**Afonso et al. 2012**	Yoga + 500 mg Calcium supplement	1. Passive stretching + 500 mg Calcium supplement; 2. Waiting List + 500 mg Calcium supplement	50–65 years	100	other mental disorder not excluded	DSM-IV	Self-report questionnaires
**Cornu et al. 2010**	Dietary supplement (soft gelatine capsules)	Placebo	25–65 years	68	excluded	DSM-IV, ICSD 2	Self-report questionnaires + Sleep diaries + Actigraphy
**Cortesi et al. 2012**	Melatonin	1.CBT-I; 2. CBT-I+melatonin; 3. Placebo	4–10 years	17.75	partially excluded	SOL and WASO > 30min on 3 or more nights a week	Self-report questionnaires + Actigraphy
**D’Aurea et al. 2019**	Resistance exercise training	1. Stretching; 2. Non-intervention	n.r.	n.r.	partially excluded	DSM-IV	Self-report questionnaires + Actigraphy + Polysomnography
**Dorn, 2000**	Valerian	Oxazepam	18–70 years	69.55	partially excluded	ICD-10	Self-report questionnaires
**Friedman et al. 2009**	1. Sleep hygiene + bright morning light 10.000lux; 2. Sleep hygiene + bright evening light 4000lux	1. Sleep hygiene + dim morning light 50lux; 2. Sleep hygiene + dim evening light 50lux	54–78 years	60	partially excluded	ICSD	Self-report questionnaires + Sleep diaries + Actigraphy + Polysomnography
**Garcia et al. 2018**	Mindfulness and relaxation training for insomnia	1. Placebo; 2. Waiting List	50–65 years	100	partially excluded	DSM-V	Self-report questionnaires + Polysomnography
**Garland et al. 2014**	Mindfulness-Based Stress Reduction	CBT-I	35–88 years	70.5	other somatic disorder not excluded	Research diagnostic criteria for insomnia, DSM-IV and ICSD	Self-report questionnaires + Sleep diaries + Actigraphy
**Gringras et al. 2012**	Melatonin	Placebo	3–15.5 years	33.5	not excluded	SOL > 60min in three nights out of five or TST < 6h in three nights out of five as reported by parents	Self-report questionnaires + Sleep diaries + Actigraphy
**Gringras et al. 2017**	Melatonin	Placebo	2–17.5 years	26.35	not excluded	DSM-V	Self-report questionnaires + Sleep diaries + Actigraphy
**Gross et al. 2011**	Mindfulness-Based Stress Reduction	Eszopiclone (LUNESTA™)	21–65 years	72.5	partially excluded	DSM-IV-TR and ICSD-2	Self-report questionnaires + Sleep diaries + Actigraphy
**Hartescu et al. 2015**	Moderate intensity physical activity	Waiting List	n.r. (>40)	73	partially excluded	Research Diagnostic Criteria	Self-report questionnaires + Sleep diaries + Actigraphy
**Hartescu et al. 2019**	Brisk walking	Waiting List	n.r.	73.2	partially excluded	Research Diagnostic Criteria	Self-report questionniares
**Huang et al. 2018**	rTMS	sham rTMS	n.r.	50	partially excluded	DSM-IV	Self-report questionnaires
**Irwin et al. 2017**	Tai Chi	CBT-I	42–83 years	100	partially excluded	DSM-4-TR and ICSD II	Self-report questionnaires + Sleep diaries + Polysomnography
**Irwin et al. 2014**	Tai Chi	1. CBT-I; 2. Sleep hygiene seminar	55–85 years	71.53	partially excluded	DSM-IV, ICSD	Self-report questionnaires + Sleep diaries + Polysomnography
**James et al. 2019**	Individualized homeopathic therapy	Placebo	n.r.	51.5	n.r.	ICD-10	Self-report questionnaires + Sleep diaries
**Jiang et al. 2013**	rTMS	1. CBT-I; 2. Pharmacotherapy	n.r.	55.5	partially excluded	DSM-IV	Self-report questionnaires + Polysomnography
**Lack et al. 2007**	Bright light 2500lux	Dim red light 100lux	18–56 years	68.75	n.r.	SOL > 45min, <30min WASO, difficulty waking spontaneously at the desired time, daytime symptoms	Self-report questionnaires + Actigraphy
**Lam, 2018**	Hypnotherapy with disease-specific suggestions	Hypnotherapy with generic suggestions	n.r.	78.5	partially excluded	DSM-V	Self-report questionnaires + Sleep diaries
**Luthringer et al. 2009**	Melatonin	Placebo	55–68 years	40	partially excluded	DSM-IV	Self-report questionnaires + Polysomnography
**Mayer et al. 2009**	Ramelteon	Placebo	18–79 years	63.2	other mental disorder not excluded	SOL>45min or TST<6,5h or difficulty initiating/maintaining sleep or nonrestorative sleep or significant impairment due to insomnia	Sleep diaries + Polysomnography
**Morin et al. 2005**	1. Valerian-hops combination; 2. Diphenhydramine	Placebo	25–65 years	59.77	excluded	SOL>30min or WASO>30min min 2 nights max 4 nights a week	Self-report questionnaires + Sleep diaries + Polysomnography
**Ong et al. 2014**	Mindfulness-based therapy for insomnia	1. Mindfulness-Based Stress Reduction; 2. Self monitoring	n.r.	73.80	partially excluded	Research Diagnostic Criteria for Insomnia Disorder	Self-report questionnaires + Sleep diaries + Actigraphy + Polysomnography
**Ong et al. 2018**	1. Mindfulness-Based Stress Reduction; 2. Mindfulness-based therapy for insomnia	Self-monitoring	n.r.	72.67	other mental or somatic disorder not excluded	Schedule for Sleep Disorders (Edinger et al. 2011) and ICSD-2	Self-report questionnaires
**Palmieri et al. 2017**	Herbal compound	Placebo	43–65.5 years	54.25	partially excluded	DSM-IV	Self-report questionnaires
**Passos et al. 2010**	1. Moderate-intensity aerobic exercise; 2. High intensity aerobic exercise; 3. Moderate intensity resistance exercise	Waiting List	30–55 years	79.15	partially excluded	DSM-IV and ICSD-2	Sleep diaries + Polysomnography
**Poyares et al. 2002**	Valerian	1. Placebo; 2. Healthy controls	n.r.	79	partially excluded	DSM-IV	Sleep diaries + Polysomnography
**Reid et al. 2010**	Aerobic physical activity + sleep hygiene	Non-physical activity + sleep hygiene	>55 years	92.85	partially excluded	difficulty falling asleep and/or staying asleep, impairment in daytime functioning, SEI<80% or awakening earlier than 6AM or sleep less then 6,5h	Self-report questionnaires
**Rodenbeck et al. 1998**	Valerian	Placebo	n.r.	n.r.	partially excluded	ICSD I	Polysomnography
**Rondanelli et al. 2011**	Melatonin	Placebo	n.r.	62.5	partially excluded	DSM-IV	Self-report questionnaires
**Roth et al. 2006**	1. Ramelteon 4mg; 2. Ramelteon 8mg	Placebo	64–93 years	58.9	partially excluded	DSM-IV-TR	Sleep diaries
**Smits et al. 2001**	Melatonin	Placebo	7–13 years	29	partially excluded	consistent with DSM-IV	Actigraphy
**Tan et al. 2016**	Exercise	Waiting List	30–65 years	0	partially excluded	DSM-IV-TR	Self-report questionnaires + Actigraphy
**van Geijlswijk et al. 2010**	Melatonin: 1. 0,5 mg/kg; 2. 0,1 mg/kg; 3. 0,15 mg/kg	Placebo	6–12 years	56.75	partially excluded	DSM-IV	Actigraphy
**Wade et al. 2007**	Melatonin	Placebo	55–80 years	39.5	excluded	DSM-IV and ICD-10	Self-report questionnaires
**Wang-Weigand et al. 2011**	Ramelteon	Placebo	18–64 years	64.7	partially excluded	DSM-IV-TR	Polysomnography
**Yeung et al. 2018**	Zero time exercise	Sleep hygiene	n.r.	91.8	partially excluded	DSM-IV	Self-report questionnaires + Actigraphy
**Zammit et al. 2007**	Ramelteon: 16 mg and 8 mg	Placebo	18–64 years	32	excluded	DSM-IV-TR	Sleep diaries + Polysomnography
**Zhang et al. 2015**	Mindfulness-Based Stress Reduction	Waiting List	>75 years	29.06	partially excluded	DSM-IV	Self-report questionnaires

Legend to Table 1: Legend: rTMS, repetitive transcranial magnetic stimulation; CBT-I, Cognitive Behavioral Therapy for Insomnia; n.r., not reported; GAD, Generalized Anxiety Disorder; DSM, Diagnostic and Statistical Manual of Mental Disorders; ICSD, International Classification of Sleep Disorders; SOL, Sleep Onset Latency; WASO, Wake After Sleep Onset; ICD, International Classification of Diseases; TST, Total Sleep Time; SEI, Sleep Efficiency Index; SD, Standard Deviation; MBSR, Mindfulness-Based Stress Reduction; MBTI, Mindfulness-Based Therapy for Insomnia; MAE, Moderate-intensity aerobic exercise; HAE, High intensity aerobic exercise; MRE, Moderate intensity resistance exercise; SHS, Sleep hygiene seminar.

## Supplementary Materials

The following are available online at https://www.mdpi.com/2077-0383/9/6/1949/s1. Document S1: PRISMA check-list, Document S2: Data extraction, Document S3: Study characteristics, Document S4: Detailed risk of bias evaluation, Document S5: Net heat plot.
