# Supplementary material for: A Systematic Review and Network Meta-Analysis of Randomized Controlled Trials Evaluating the Evidence Base of Melatonin, Light Exposure, Exercise, and Complementary and Alternative Medicine for Patients with Insomnia Disorder
Source: J Clin Med. 2020 Jun 22;9(6):1949. doi: 10.3390/jcm9061949 (PMC7356922; doi:10.3390/jcm9061949)
Supplement: Supplementary file 1 [file jcm-09-01949-s001.zip › jcm-827728-supplementary/S5_Netheat.pdf]

subjective sleep quality

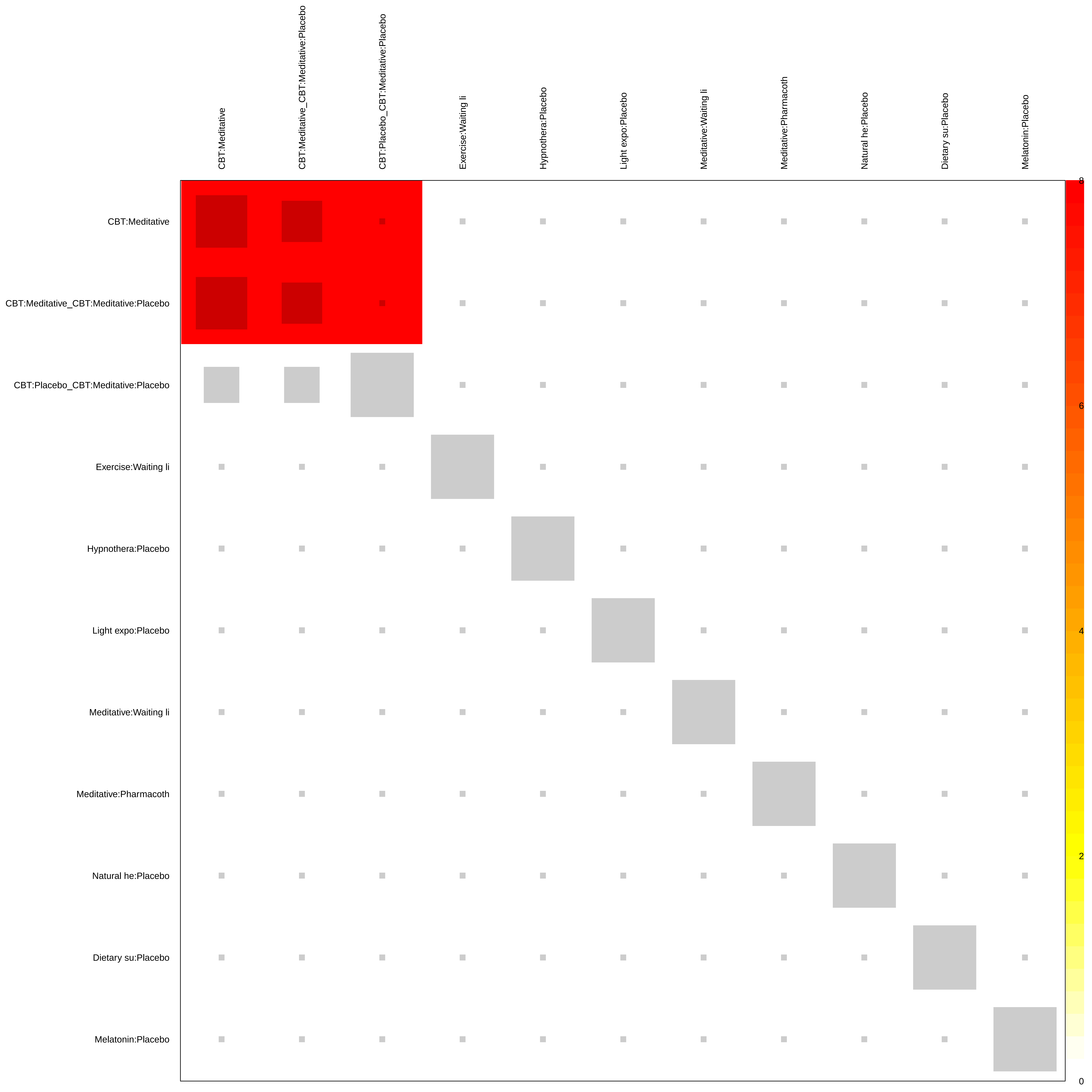
CBT:Meditative
CBT:Meditative_CBT:Meditative:Placebo
CBT:Placebo_CBT:Meditative:Placebo
Exercise:Waiting li
Hypnothera:Placebo
Light expo:Placebo
Meditative:Waiting li
Meditative:Pharmacoth
Natural he:Placebo
Dietary su:Placebo
Melatonin:Placebo
8
6
4
2
0

objective sleep quality

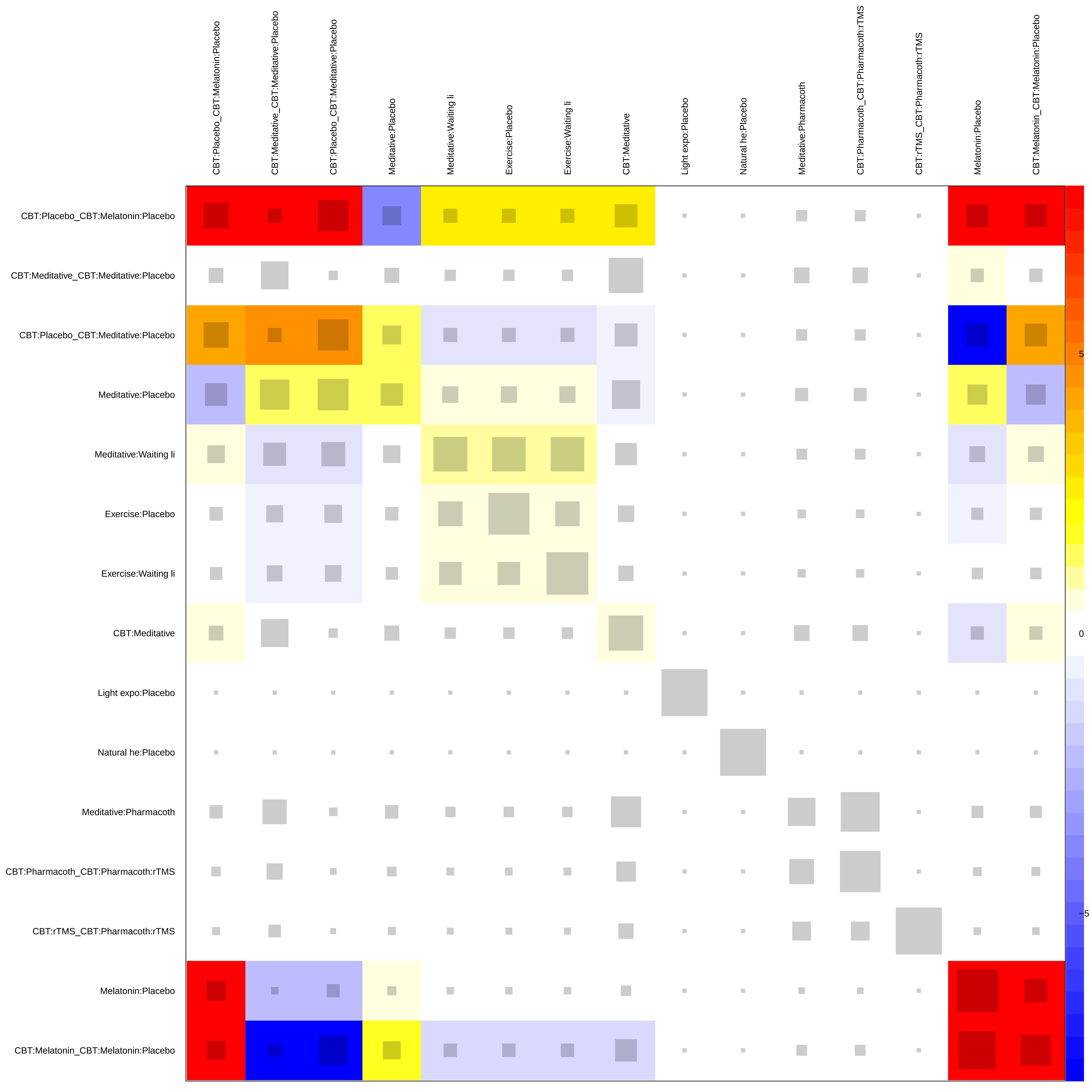
CBT:Placebo_CBT:Melatonin:Placebo
CBT:Meditative_CBT:Meditative:Placebo
CBT:Placebo_CBT:Meditative:Placebo
Meditative:Placebo
Meditative:Waiting li
Exercise:Placebo
Exercise:Waiting li
CBT:Meditative
Light expo:Placebo
Natural he:Placebo
Meditative:Pharmacoth
CBT:Pharmacoth_CBT:Pharmacoth:rTMS
CBT:rTMS_CBT:Pharmacoth:rTMS
Melatonin:Placebo
CBT:Melatonin_CBT:Melatonin:Placebo
5
0
−5

sleepiness

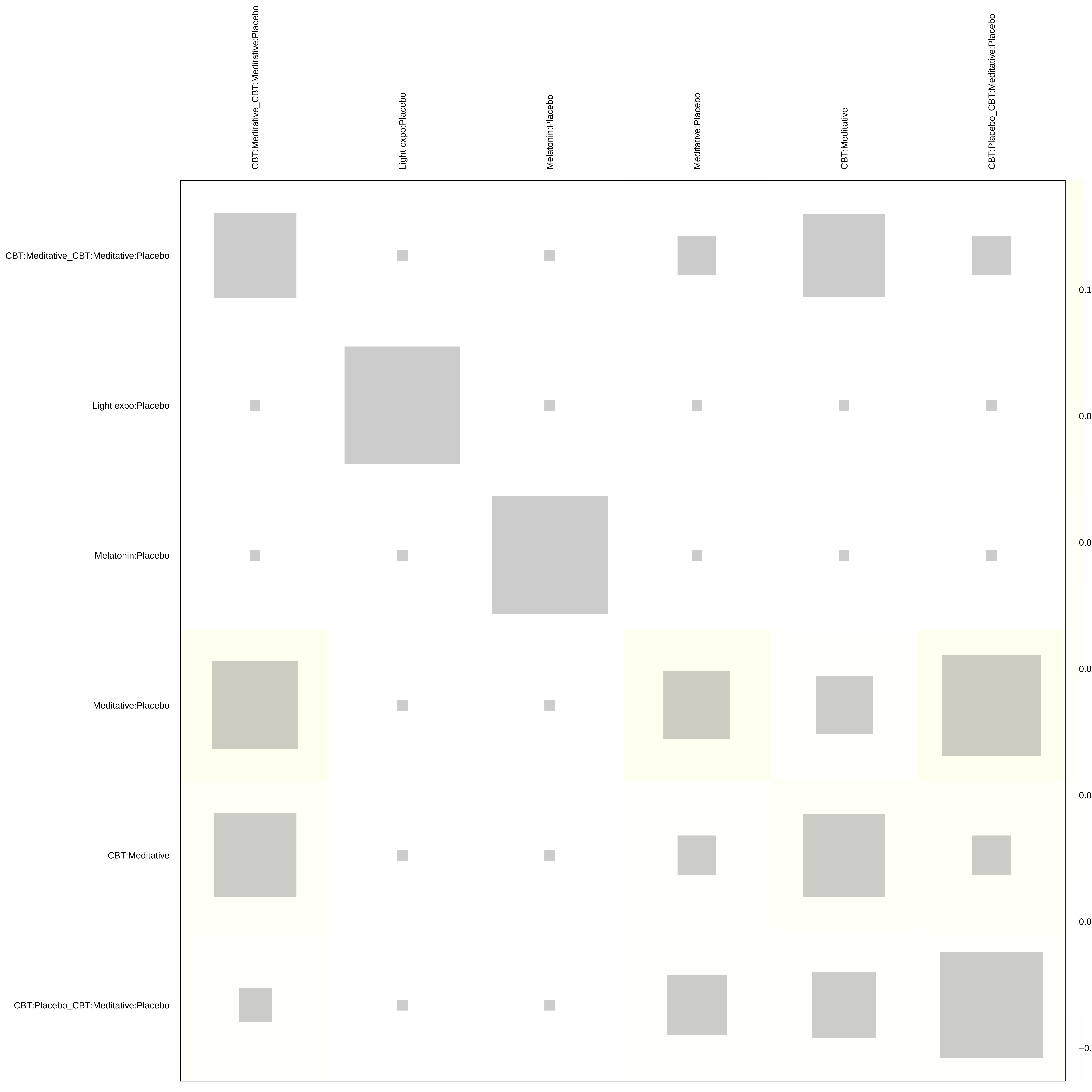

CBT:Meditative_CBT:Meditative:Placebo
Light expo:Placebo
Melatonin:Placebo
Meditative:Placebo
CBT:Meditative
CBT:Placebo_CBT:Meditative:Placebo
CBT:Meditative_CBT:Meditative:Placebo
Light expo:Placebo
Melatonin:Placebo
Meditative:Placebo
CBT:Meditative
CBT:Placebo_CBT:Meditative:Placebo
0.1
0.0
0.0
0.0
0.0
0.0
−0.

subjective severity of sleep problem

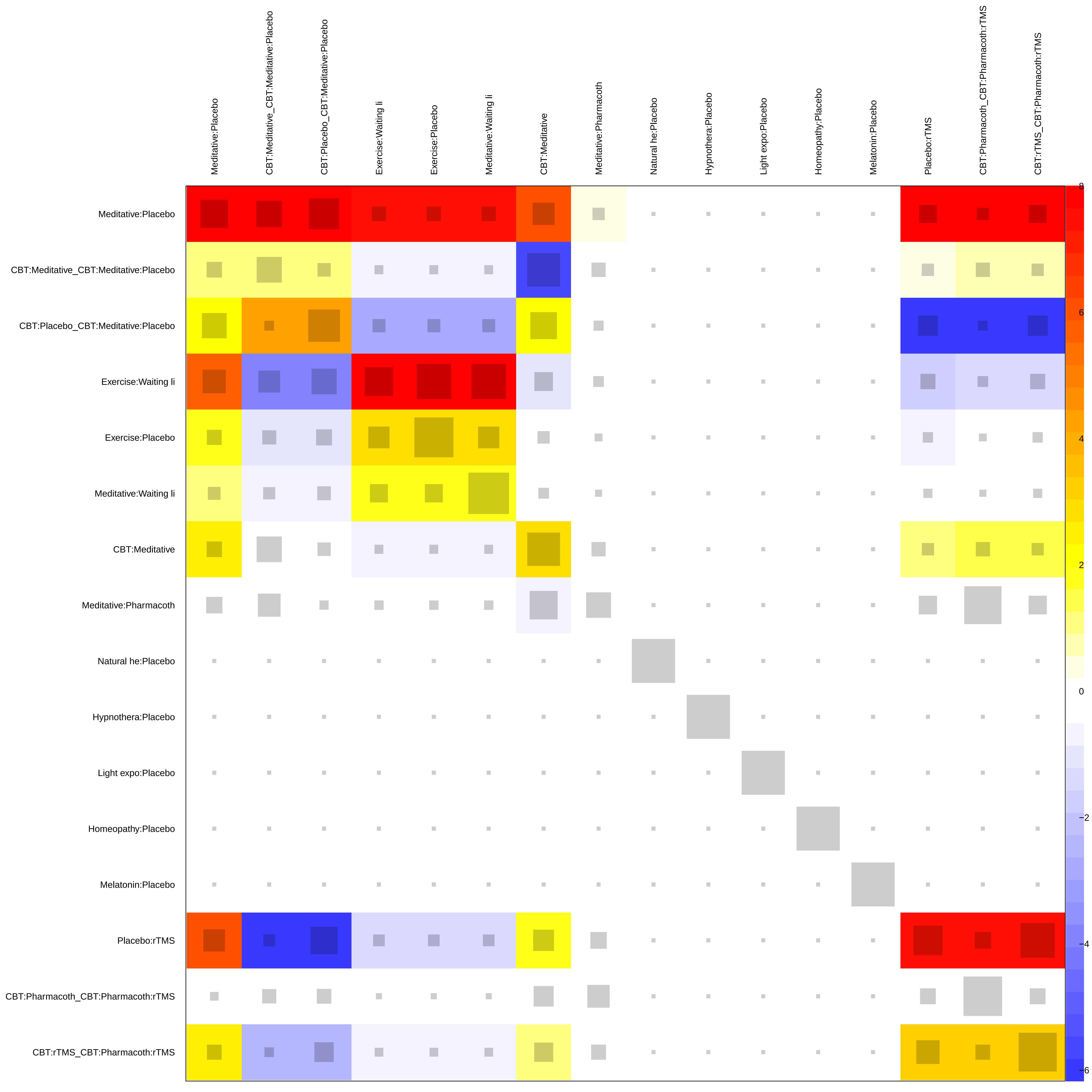

Meditative:Placebo
CBT:Meditative_CBT:Meditative:Placebo
CBT:Placebo_CBT:Meditative:Placebo
Exercise:Waiting li
Exercise:Placebo
Meditative:Waiting li
CBT:Meditative
Meditative:Pharmacoth
Natural he:Placebo
Hypnothera:Placebo
Light expo:Placebo
Homeopathy:Placebo
Melatonin:Placebo
Placebo:rTMS
CBT:Pharmacoth_CBT:Pharmacoth:rTMS
CBT:rTMS_CBT:Pharmacoth:rTMS
8
6
4
2
0
−2
−4
−6

subjective sleep onset

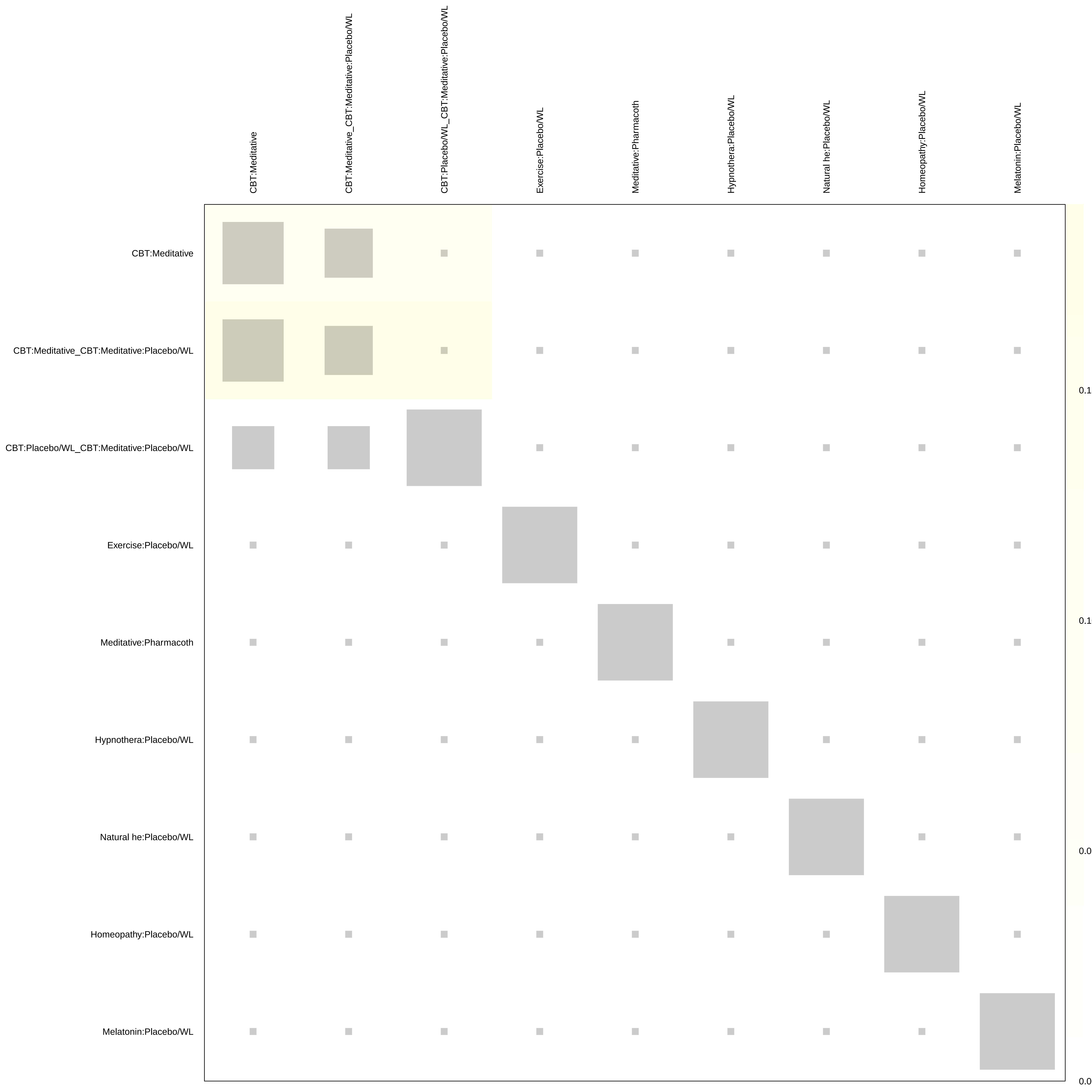

CBT:Meditative
CBT:Meditative_CBT:Meditative:Placebo/WL
CBT:Placebo/WL_CBT:Meditative:Placebo/WL
Exercise:Placebo/WL
Meditative:Pharmacoth
Hypnothera:Placebo/WL
Natural he:Placebo/WL
Homeopathy:Placebo/WL
Melatonin:Placebo/WL
CBT:Meditative
CBT:Meditative_CBT:Meditative:Placebo/WL
CBT:Placebo/WL_CBT:Meditative:Placebo/WL
Exercise:Placebo/WL
Meditative:Pharmacoth
Hypnothera:Placebo/WL
Natural he:Placebo/WL
Homeopathy:Placebo/WL
Melatonin:Placebo/WL
0.1
0.1
0.0
0.0

objective sleep onset

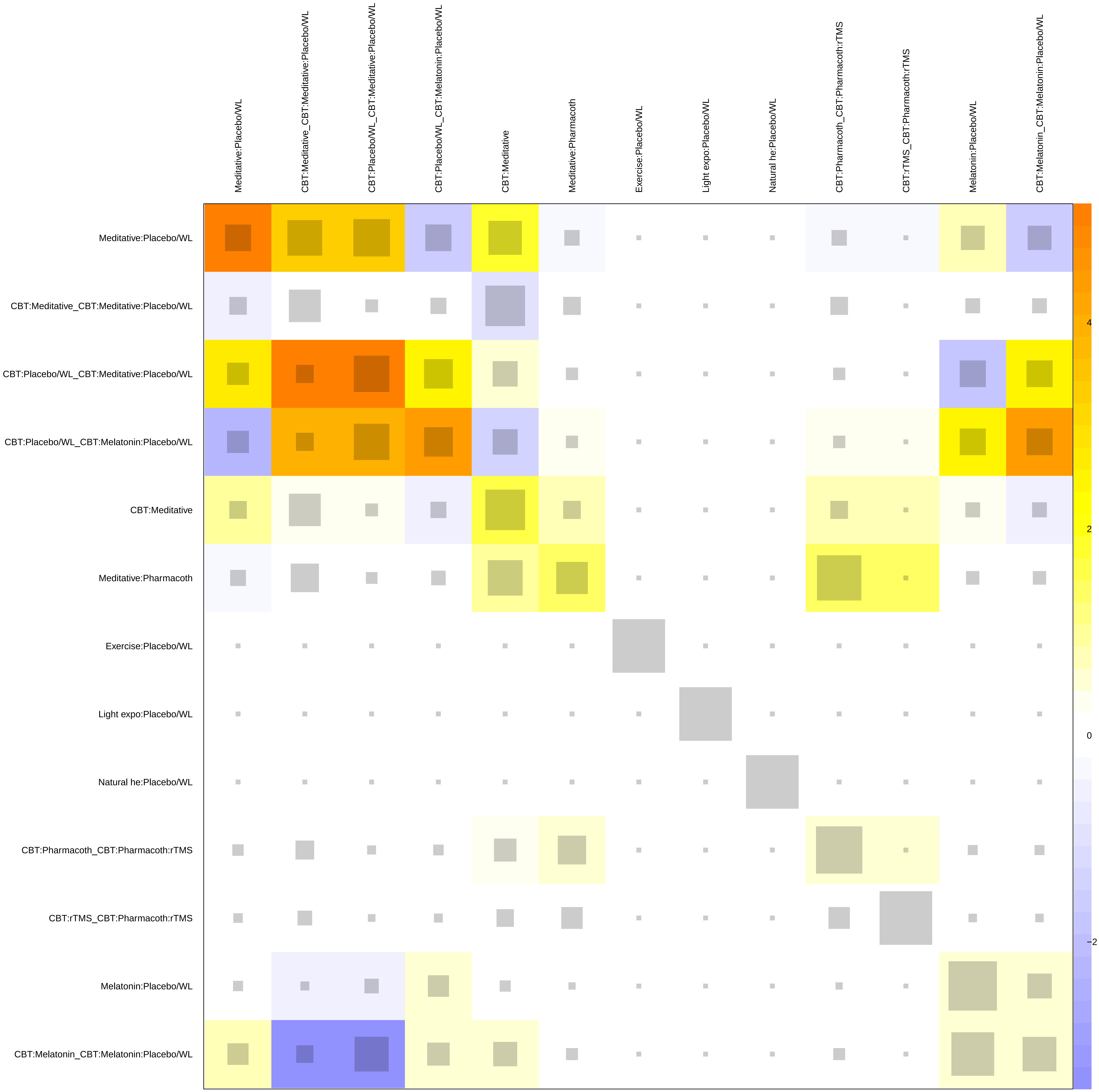
Meditative:Placebo/WL
CBT:Meditative_CBT:Meditative:Placebo/WL
CBT:Placebo/WL_CBT:Meditative:Placebo/WL
CBT:Placebo/WL_CBT:Melatonin:Placebo/WL
CBT:Meditative
Meditative:Pharmacoth
Exercise:Placebo/WL
Light expo:Placebo/WL
Natural he:Placebo/WL
CBT:Pharmacoth_CBT:Pharmacoth:rTMS
CBT:rTMS_CBT:Pharmacoth:rTMS
Melatonin:Placebo/WL
CBT:Melatonin_CBT:Melatonin:Placebo/WL
4
2
0
−2

subjective wake after sleep onset

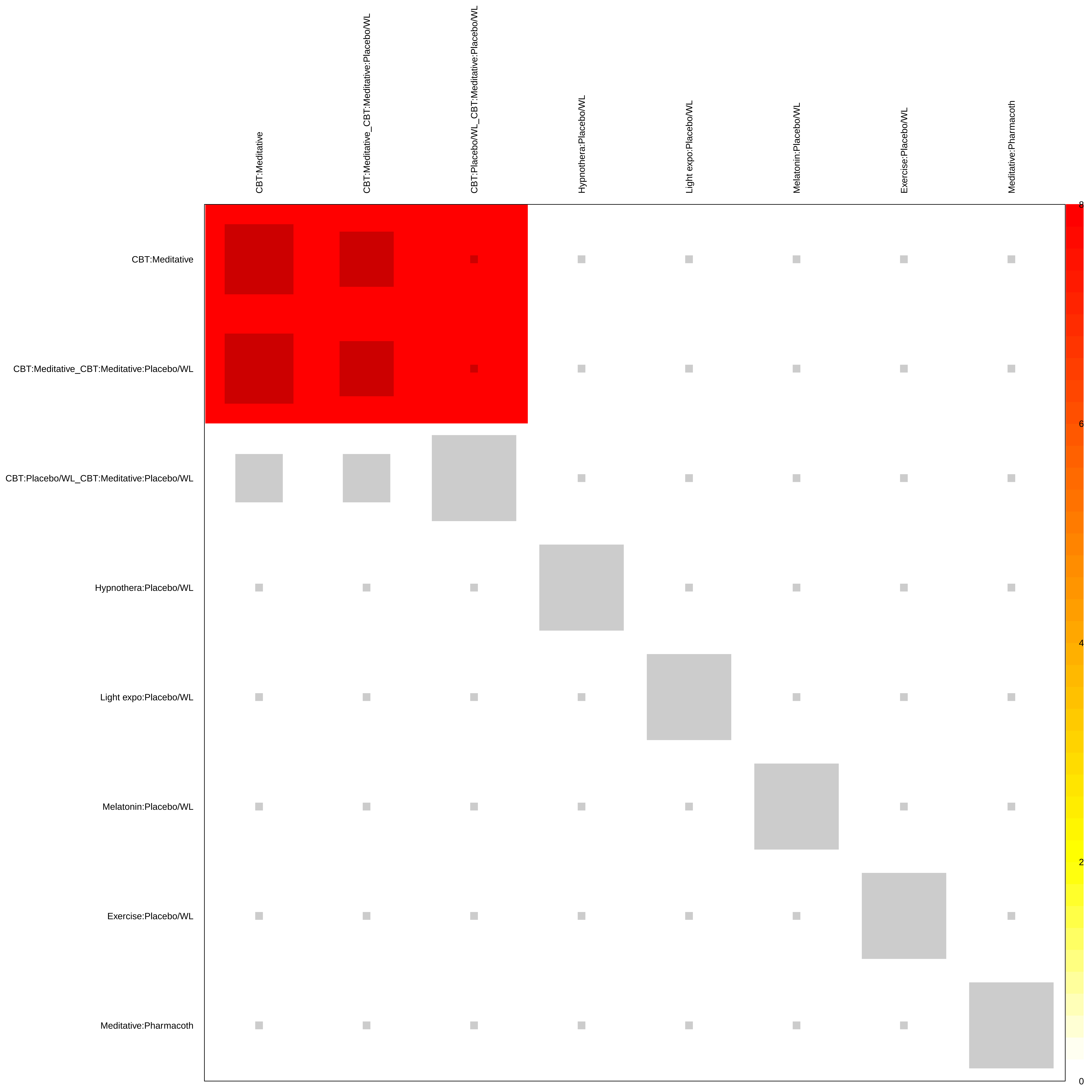

CBT:Meditative
CBT:Meditative_CBT:Meditative:Placebo/WL
CBT:Placebo/WL_CBT:Meditative:Placebo/WL
Hypnothera:Placebo/WL
Light expo:Placebo/WL
Melatonin:Placebo/WL
Exercise:Placebo/WL
Meditative:Pharmacoth
8
6
4
2
0

objective wake after sleep onset

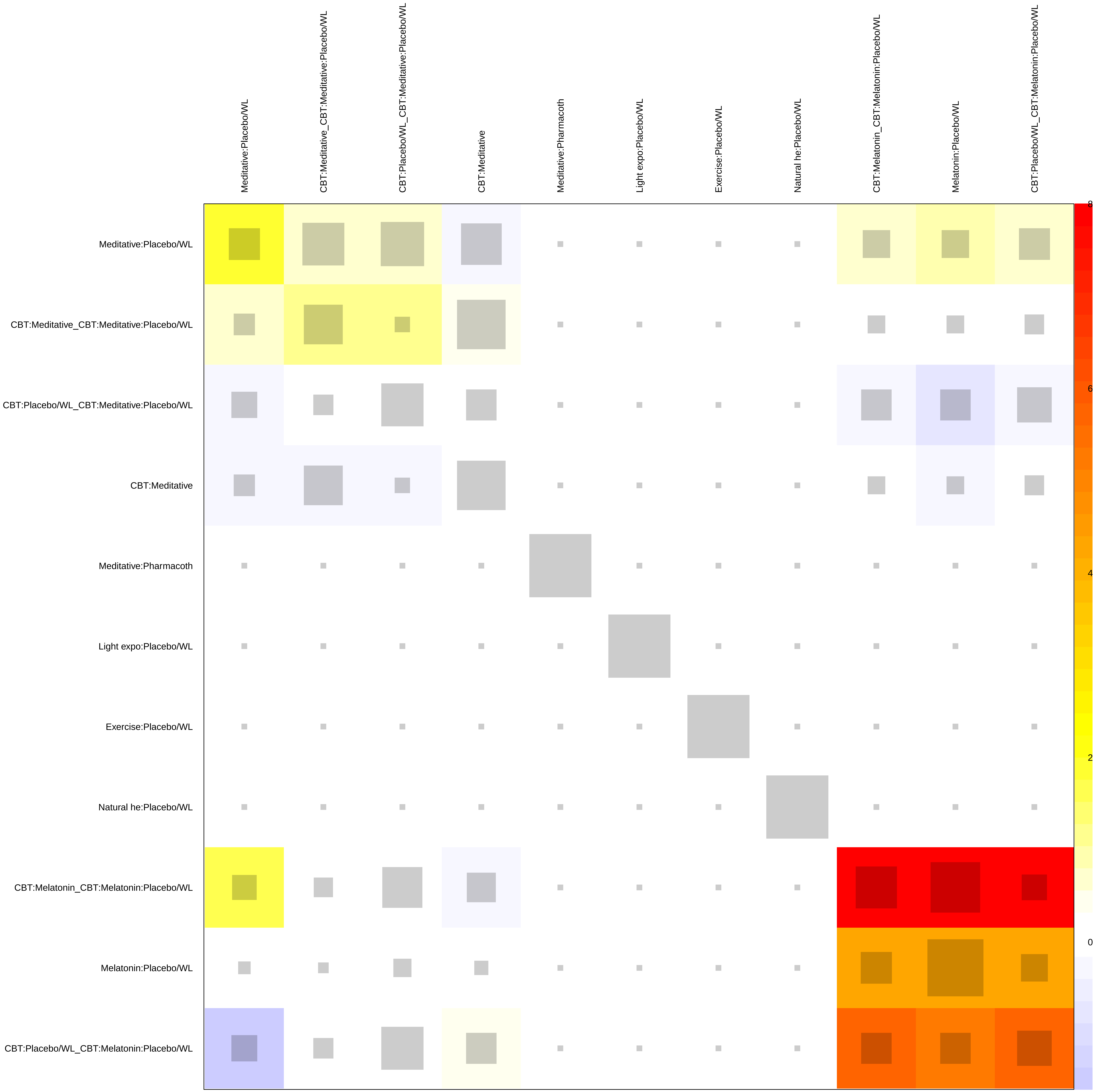
Meditative:Placebo/WL
CBT:Meditative_CBT:Meditative:Placebo/WL
CBT:Placebo/WL_CBT:Meditative:Placebo/WL
CBT:Meditative
Meditative:Pharmacoth
Light expo:Placebo/WL
Exercise:Placebo/WL
Natural he:Placebo/WL
CBT:Melatonin_CBT:Melatonin:Placebo/WL
Melatonin:Placebo/WL
CBT:Placebo/WL_CBT:Melatonin:Placebo/WL
Meditative:Placebo/WL
CBT:Meditative_CBT:Meditative:Placebo/WL
CBT:Placebo/WL_CBT:Meditative:Placebo/WL
CBT:Meditative
Meditative:Pharmacoth
Light expo:Placebo/WL
Exercise:Placebo/WL
Natural he:Placebo/WL
CBT:Melatonin_CBT:Melatonin:Placebo/WL
Melatonin:Placebo/WL
CBT:Placebo/WL_CBT:Melatonin:Placebo/WL
8
6
4
2
0
